# Novel Visceral Obesity Indicators and Associated Metabolic Fingerprint in Incident Diabetic Retinopathy

**DOI:** 10.1167/iovs.66.12.17

**Published:** 2025-09-08

**Authors:** Yingcheng He, Jingxin Zhou, Jiawei Wang, Shenyu Huang, Huimin Li, Jing Cao, Juan Ye

**Affiliations:** 1 Eye Center, The Second Affiliated Hospital, School of Medicine, Zhejiang University, Zhejiang Provincial Key Laboratory of Ophthalmology. Zhejiang Provincial Clinical Research Center for Eye Diseases, Zhejiang Provincial Engineering Institute on Eye Diseases, Hangzhou, People's Republic of China

**Keywords:** diabetic retinopathy (DR), visceral obesity, risk factor, UK Biobank (UKB), metabolism

## Abstract

**Purpose:**

Evidence on the association between visceral obesity and diabetic retinopathy (DR) remains sparse and debatable. We aimed to use three novel indicators, body roundness index (BRI), lipid accumulation product (LAP), and visceral adiposity index (VAI), to investigate the longitudinal relationship between visceral obesity and DR, and explore the potential metabolic mechanisms.

**Methods:**

In this prospective study based on the UK Biobank (UKB), 14,738 individuals with diabetes free of DR at baseline were included. Cox proportional hazards regression models were adopted to explore the association among the three indices and DR. Based on nuclear magnetic resonance (NMR) metabolomics of 168 plasma metabolites, we performed elastic net regression to select metabolites associated with visceral obesity and DR. Enrichment and pathway analyses were used to investigate biological pathways and network analysis was performed to discover the metabolic interactions.

**Results:**

Over a 10-year follow-up, 1594 subjects developed DR. In the fully adjusted models, the three indices were significantly correlated with a high risk of DR, especially in Caucasians. Furthermore, we identified a metabolic fingerprint comprising 12 biomarkers associated with visceral obesity and DR, including 3-hydroxybutyrate, acetone, alanine, albumin, citrate, intermediate-density lipoprotein particles, creatinine, glucose, glycine, glycoprotein acetyls, pyruvate, and tyrosine. These metabolites were mainly enriched in alanine metabolism and their interaction was partly through succinic acid.

**Conclusions:**

Visceral obesity indices (BRI, LAP, and VAI) were positively associated with DR onset in patients with DM, and the potential mechanism involves alanine and energy metabolism. Our findings may promote early detection and precision prevention of DR.

As a crucial microvascular complication of diabetes mellitus (DM), diabetic retinopathy (DR) impacts approximately one-third of patients with DM, remaining one of the leading causes of blindness worldwide.^1^^,^^2^ Global age-standardized prevalence of blindness caused by DR has risen from 11.4% in 1990 to 18.4% in 2020, and DR is projected to impact over 160 million people by 2045.^3^^,^^4^ Although tight control of glucose and blood pressure is the primary way to delay DR onset, growing evidence shows this approach is insufficient, highlighting the importance of finding other modifiable risk factors.^5^^–^^7^

Obesity is an essential risk factor of DM, but its relationship with DR remains controversial.^8^^–^^10^ Visceral obesity, a stronger predictor of DM than general obesity, may also play a role in DR development, although this relationship remains unclear due to limited evidence.^11^^,^^12^ Visceral obesity refers to the type of obesity characterized by excessive accumulation of fat in the abdominal organs.^13^ Nevertheless, the benchmark assessment of visceral obesity involves measuring visceral adipose tissues (VATs) through magnetic resonance imaging (MRI) or computed tomography (CT), which is time-consuming and costly.^14^ Hence, a variety of visceral obesity indices have been proposed, among which waist circumference (WC), waist-to-hip ratio (WHR), and body mass index (BMI) are the classical ones.^15^ However, these indices were relatively rough and ignored the influence of serum metabolites on obesity status.^16^^,^^17^ Thus, new indices have come out. Visceral adiposity index (VAI), incorporating serum lipids, BMI, and WC, serves as a marker of visceral adipose function and correlates with DM occurrence.^18^ Based on WC and triglycerides (TGs), lipid accumulation product (LAP) is also an indicator of central fat accumulation and can predict insulin resistance.^19^^,^^20^ Body roundness index (BRI), derived from height and WC, is an established predictor for VAT percentage.^21^^,^^22^

Metabolomics is a large-scale study of quantifying a great number of metabolites in a single measurement, thus timely detecting the fluctuation of biological pathways.^23^ Researchers have utilized serum metabolomic data to predict the risk of DM and DR, demonstrating its great promise in disease prediction.^24^^–^^26^ Metabolic information can also be used to investigate the mechanism by which risk factors influence diseases. Specifically, lifestyle-related metabolites proved to enhance the prediction of coronary heart disease.^27^ Visceral obesity was strongly associated with plasma metabolites such as TG-rich lipoproteins and docosahexaenoic acid.^28^^,^^29^ Therefore, visceral adiposity may induce DR onset by impacting metabolomic change. Despite the potential, this topic currently lacks research.

Herein, we aimed to explore the longitudinal association among the three established indices of visceral obesity (BRI, LAP, and VAI) and incident DR based on approximately 15,000 patients with DM in the UK Biobank (UKB). Further, by leveraging serum metabolomic data from a high-throughput targeted nuclear magnetic resonance (NMR) metabolomics platform in the UKB, we investigated the metabolic mechanism of visceral obesity in DR onset.

## Methods

### Study Population

The UKB is a prospective cohort study that recruited over 0.5 million participants aged 40 to 69 years from 22 assessment centers across the United Kingdom. The initial assessment was conducted from 2006 to 2010, encompassing touchscreen questionnaires, verbal interviews, physical measurements, biological sampling, and electronic health records. The detailed methodology was described elsewhere.^30^^,^^31^

**Figure 1.**
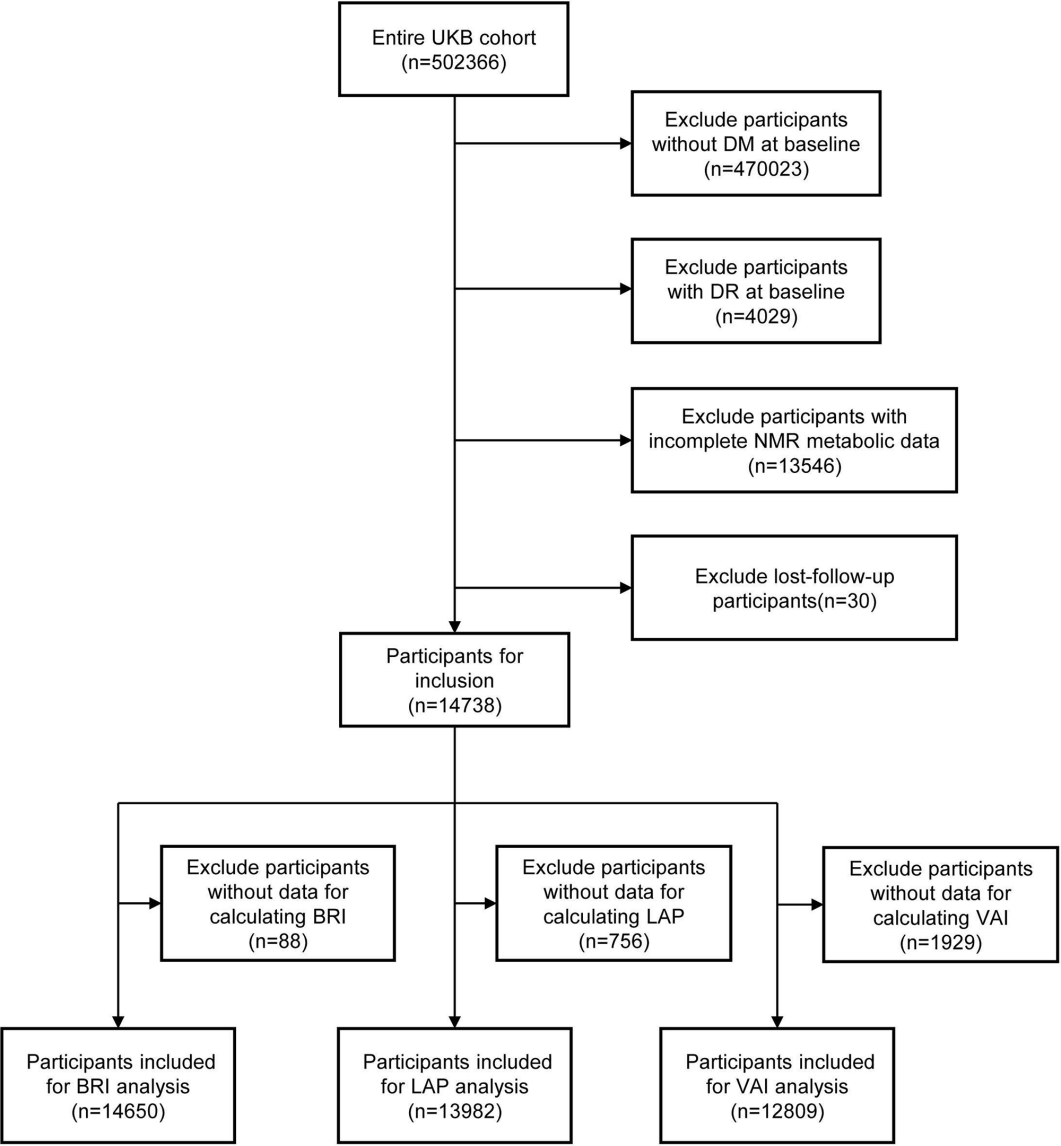
**The flowchart of screening subjects.** UKB, UK Biobank; DM, diabetes mellitus; DR, diabetic retinopathy; NMR, nuclear magnetic resonance; BRI, body roundness index; LAP, lipid accumulation product; VAI, visceral adiposity index.

From the original 502,366 subjects, we retained 32,343 individuals with prevalent DM (defined as DM diagnosed before the date of baseline assessment). Then, 4029 people with DR at baseline (defined as DR diagnosed before the date of baseline assessment) were excluded. We also eliminated those individuals with incomplete NMR metabolomic data or those lost to follow-up. In total, 14,738 participants with DM free from DR were included in the primary study. Based on this, we excluded individuals without complete data to calculate BRI, LAP, and VAI. Therefore, 14,650 subjects with BRI, 13,982 subjects with LAP, and 12,809 subjects with VAI were included in the current study. The study workflow is illustrated in Figure 1.

### Definitions of DM and DR

DM was ascertained by (1) self-reported diagnosis of DM or hypoglycemic medication use during verbal interviews, (2) physician diagnosis or insulin use recorded in touchscreen questionnaires, (3) hospital admission data of DM diagnosis, (4) information on the first occurrences of DM onset, and (5) glycated hemoglobin (HbA1c) level ≥48 mmol/mol (6.5%) or random glucose level ≥11.1 mmol/L.^32^ The diagnosis of DR was derived from (1) self-reported diagnosis of DR in touchscreen questionnaires, (2) hospital admission data of DR diagnosis, and (3) information on the first occurrences of DR onset. The International Classification of Diseases, 10th Revision (ICD-10) was used for diagnosis. The field IDs and codes for diagnosis are shown in Supplementary Tables S1 and S2.

### Visceral Obesity Indices

The three indices of visceral obesity are BRI, LAP, and VAI. Trained personnel conducted the anthropometric measurements at baseline using standardized protocols. WC, hip circumference (HC), standing height, and body weight were measured. BMI is defined as one's weight (in kilograms) divided by the square of the height (in meters). This index is categorized into four levels: underweight (<18.5 kg/m^2^), normal weight (18.5 ≤ BMI <25 kg/m^2^), overweight (25 ≤ BMI <30 kg/m^2^), or obesity (≥30 kg/m^2^).^33^ WHR is the ratio of WC to HC. At recruitment, blood was sampled for a variety of tests, including blood biochemistry assays. The levels of TG and high-density lipoprotein cholesterol (HDL-C) were used to calculate visceral obesity indices.

The formula of BRI is shown below^21^:

$$\begin{array}{c} BRI=364.2-365.5\;\times \sqrt{1-{\left(\frac{WC\left(cm\right)/2\pi }{0.5\times height\left(cm\right)}\right)}^{2}} \end{array}$$

The formula of LAP appears as follows^20^:

$$\begin{array}{c} Female\;LAP=\left[WC\left(cm\right)-58\right]\times TG\left(mmol/L\right) \end{array}$$

$$\begin{array}{c} Male\;LAP=\left[WC\left(cm\right)-65\right]\times TG\left(mmol/L\right) \end{array}$$

The formula of VAI is presented below^18^:

$$\begin{array}{ccc} & & \;Female\;VAI=\left(\frac{WC\left(cm\right)}{36.58+1.89\;\times BMI\left(kg/m^{2}\right)}\right)\\ & & \;\times \left(\frac{TG\left(mmol/L\right)}{0.81}\right)\times \left(\frac{1.52}{HDL\left(mmol/L\right)}\right) \end{array}$$

$$\begin{array}{ccc} & & \;Male\;VAI=\left(\frac{WC\left(cm\right)}{39.68+1.88\;\times BMI(kg/m^{2})}\right)\\ & & \;\times \left(\frac{TG\left(mmol/L\right)}{1.03}\right)\times \left(\frac{1.31}{HDL\left(mmol/L\right)}\right) \end{array}$$

### Covariates

Age and sex were self-reported. Socioeconomic status was evaluated using the Townsend deprivation index (TDI), an area-based metric derived from residential postal codes.^34^ Education was categorized into two levels, depending on the achievement of a college or university degree. Lifestyle factors such as smoking and alcohol status were self-reported and were divided into never, previous, and current.

Furthermore, we collected various factors associated with DM, including hypertension, HbA1c and glucose level, ethnicity, duration, and family history of diabetes. Systolic blood pressure (SBP) and diastolic blood pressure (DBP) were recorded as the mean of two readings based on standardized protocols, respectively. Hypertension was determined by SBP over 140 millimeters of mercury (mm Hg) or DBP over 90 mm Hg. Duration of diabetes was defined as the period from the onset of diabetes to the study baseline. We ascertained a family history of diabetes by the occurrence of DM in parents or siblings. We integrated the medication information from verbal interviews and questionnaires to obtain the diabetic medication use, which was categorized into four types: oral hypoglycemic agents, cholesterol-lowering agents, antihypertensive agents, and insulin. The serum creatinine, HDL, low-density lipoprotein (LDL), and TGs were measured by standardized blood assays. Details of UKB assessments were presented in the online protocol.^35^

### Metabolome Profiling and Quantification

Through NMR spectroscopy-derived metabolomic profiling (Nightingale Health, Helsinki, Finland), 249 metabolic biomarkers (168 directly measured biomarkers and 81 ratios) of ethylenediaminetetraacetic acid (EDTA) plasma from approximately 280,000 individuals were measured in the UKB. The detailed description of the NMR protocol was recorded in the published documents.^36^ In this study, we selected 168 directly measured biomarkers from baseline assessment, including lipids, fatty acids, ketone bodies, amino acids, lipoproteins, and glycolytic metabolites (Supplementary Table S3). All metabolite levels underwent natural log transformation (ln[X + 1]) and z-score normalization.

### Statistical Analysis

For covariates, missing values of continuous variables were replaced by medians, whereas those of categorical variables were imputed using modes. The specific missing values and ratios of the covariates were also calculated. To compare the baseline features between DR and non-DR groups, chi-squared or the independent *t*-test was used wherever appropriate. Spearman's correlation was conducted to assess the relationship among the 168 metabolites.

The association among the three visceral obesity indices and incident DR was assessed using multivariable-adjusted Cox proportional hazard regression models. We performed z-score normalization for the values of BRI, VAI, and LAP. Three models were constructed: model 1 was adjusted for age and gender; model 2 was adjusted for age, gender, education level, TDI, smoking status, and alcohol status; and model 3 was adjusted for model 2 variables plus DM risk factors, including ethnicity, hypertension, HbA1c level, duration of DM, and family history of DM. The scaled Schoenfeld residuals were used to test the proportional hazards assumptions for model fit. To capture the nonlinear relationship between visceral obesity indices and DR, we performed restricted cubic splines (RCSs) with four knots at the fifth, 35th, 65th, and 95th percentiles.

Subgroup analysis was conducted to assess the robustness of the primary results. Cox models were stratified by age (<60 and ≥60 years), gender (males and females), ethnicity (White and non-White), duration of DM (<5 and ≥5 years), BMI (normal weight, overweight, and obesity), and hypertension (yes and no). Four sensitivity analyses were also carried out. First, we excluded individuals who developed DR within 3 years of follow-up to explore potential reverse causation. Second, we fitted Fine and Gray models to test the competing effect of death on the risk of DR. Third, we added BMI and WHR to model 3 to test the influence of these indices. Finally, we performed multiple imputation by chained equations (MICEs) instead of median/mode imputation to handle the missing data and assessed the reliability of the results.

The statistical analyses were performed using R (version 4.4.1, The R Project for Statistical Computing). *P* < 0.05 was considered statistically significant.

### Metabolomics Analysis

We used elastic net (EN) regularized linear regression models to select metabolic biomarkers associated with BRI, LAP, or VAI. Additionally, an EN regularized Cox regression model was performed for screening metabolomic predictors of DR. The population was split into a training set and a validation set in a 7:3 ratio. To reach the sparsity of the model, we tested α from 0.1 to 0.9 (every 0.1 apart). Then, the EN model was constructed based on the best α and 10-fold cross-validation. Metabolites with nonzero coefficients were extracted for further analysis. The circle heatmaps of significant metabolites were drawn using ChiPlot (https://www.chiplot.online/). Metabolites associated with both visceral obesity and DR were identified through intersection analysis across the four cohorts. Enrichment and pathway analysis were carried out using MetaboAnalyst online platform (version 6.0) based on the human Kyoto Encyclopedia of Genes and Genomes (KEGG) and Small Molecule Pathway Database (SMPDB).^37^^,^^38^ The enrichment method was a hypergeometric test and the topology parameter was relative-betweenness centrality. Pathways were considered statistically significant when the *P* value < 0.05. To explore the correlation between the intersected metabolites, a metabolite-metabolite interaction network was made (https://www.metaboanalyst.ca).^39^

## Results

### Population Characteristics

The features of the study populations stratified by incident DR are described in Table 1. Among 14,738 initially DR-free DM participants, 1594 (10.8%) developed DR and 2464 (16.7%) died during an approximately 10-year follow-up. The incident DR group tended to be older, socioeconomically deprived, non-White, with higher HbA1c and glucose levels, longer DM duration, and a higher prevalence of hypertension and family history of DM. Besides, the DR group also used more medication for diabetes and had a differential serum profile characterized by higher creatinine and lower LDL. More importantly, the incident DR group had a higher prevalence of visceral obesity compared to the non-DR group, which was evidenced by elevated values in all five obesity indices. The missing ratios of several key covariates were smoking (0.14%), alcohol (0.51%), and HbA1c (4.61%; Supplementary Table S4). The features of the incident DR group remained generally consistent in the cohorts for BRI, LAP, and VAI analyses and in the cohort excluding subjects with any missing covariates (Supplementary Tables S5–S8). The ethnic composition is detailed in Supplementary Table S9.

**Table 1.** Baseline Characteristics of the Subjects

Characteristics Mean (SD) or *n* (%)	Non-DR (*N* = 13,144)	DR (N = 1,594)	*P* Value
Age, y, mean (SD)	59.00 (7.47)	60.19 (6.89)	<0.001
Male, *n* (%)	7,905 (60.1)	946 (59.3)	0.559
College or university degree, *n* (%)	3,147 (23.9)	352 (22.1)	0.106
Townsend Deprivation Index, mean (SD)	−0.57 (3.36)	−0.34 (3.47)	0.008
Smoking, *n* (%)			0.798
Never	6,144 (46.7)	732 (45.9)	
Previous	5,504 (41.9)	681 (42.7)	
Current	1,496 (11.4)	181 (11.4)	
Alcohol, n (%)			0.156
Never	1,094 (8.3)	154 (9.6)	
Previous	871 (6.6)	111 (7.0)	
Current	11,179 (85.1)	1,329 (83.4)	
Follow-up time, y, mean (SD)	12.80 (2.71)	9.04 (3.72)	<0.001
Diabetes-related factors			
White, *n* (%)	11,669 (88.8)	1,359 (85.3)	<0.001
HTN, *n* (%)	9,985 (76.0)	1,399 (87.8)	<0.001
HbA1c, mmol/mol, mean (SD)	50.82 (14.10)	57.82 (15.50)	<0.001
Glucose, mmol/L, mean (SD)	7.09 (2.95)	8.32 (3.95)	<0.001
Duration of diabetes, y, mean (SD)	6.78 (8.24)	10.01 (10.35)	<0.001
Family history of diabetes, *n* (%)	4,965 (37.8)	655 (41.1)	0.011
Diabetes medication use, *n* (%)			
Oral hypoglycemic drugs	5,882 (44.8)	978 (61.4)	<0.001
Cholesterol-lowering drugs	8,341 (63.5)	1,175 (73.7)	<0.001
Hypotensive drugs	7,324 (55.7)	1,002 (62.9)	<0.001
Insulin use	1,491 (11.3)	454 (28.5)	<0.001
Blood biochemistry markers, mean (SD)			
Serum creatinine, umol/L	73.95 (18.56)	76.23 (32.09)	<0.001
HDL cholesterol, mmol/L	1.20 (0.30)	1.19 (0.30)	0.172
LDL, mmol/L	2.90 (0.85)	2.74 (0.79)	<0.001
Triglycerides, mmol/L	2.18 (1.25)	2.22 (1.35)	0.241
Obesity indices, mean (SD)			
BMI	31.36 (5.85)	31.79 (5.89)	0.006
WHR	0.94 (0.09)	0.95 (0.09)	<0.001
BRI	5.68 (1.95)	5.95 (2.05)	<0.001
LAP	91.19 (66.12)	96.98 (74.21)	0.002
VAI	3.17 (2.35)	3.33 (2.69)	0.020

BMI, body mass index; BRI, body roundness index; DR, diabetic retinopathy; HDL, high-density lipoprotein; HTN, hypertension; LAP, lipid accumulation product; LDL, low-density lipoprotein; VAI, visceral adiposity index; WHR, waist-to-hip ratio.

Data are presented as the means ± standard deviations (SDs), numbers and (percentages).

### Association Among Three Visceral Obesity Indices and DR

**Figure 2.**
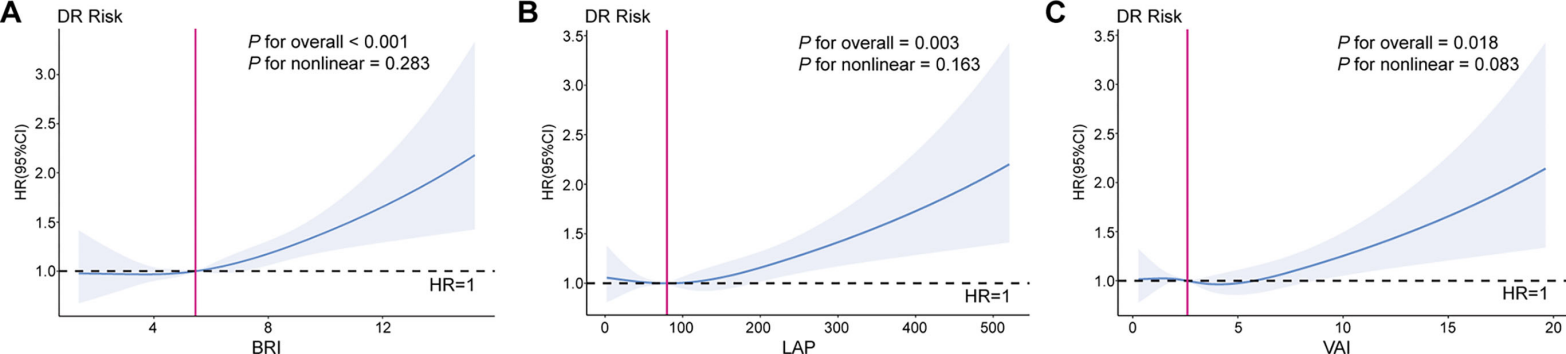
**Restricted cubic spline analysis of the relationship between visceral obesity indices and DR.** (**A**) The nonlinear relationship between body roundness index (BRI) and diabetic retinopathy (DR; *P* for overall < 0.001, and *P* for nonlinear = 0.283). (**B**) The nonlinear relationship between lipid accumulation product (LAP) and DR (*P* for overall = 0.003, and *P* for nonlinear = 0.163). (**C**) The nonlinear relationship between visceral adiposity index (VAI) and DR (*P* for overall = 0.018, and *P* for nonlinear = 0.083). DR, diabetic retinopathy; HR, hazard ratio; CI, confidence interval; BRI, body roundness index; LAP, lipid accumulation product; VAI, visceral adiposity index.

The relationships among the three visceral obesity indices and incident DR are indicated in Table 2. The mean and the standard deviation (SD) of BRI, LAP, and VAI were 0 and 1 after z-score normalization. In the fully adjusted model, BRI, LAP, and VAI were all significantly associated with incident DR. A 1-SD increment of BRI was consistently linked to a higher DR risk in all three models (hazard ratio [HR] = 1.11, 95% confidence interval [CI] = 1.06–1.17, *P* < 0.001) in model 3. Similar results were found in LAP and VAI (HR = 1.08, 95% CI = 1.03–1.13, *P* < 0.01; and HR = 1.05, 95% CI = 1.00–1.11, *P* < 0.05). Additionally, no significant nonlinear relationships were observed among the three visceral obesity indices and incident DR (*P* for nonlinear > 0.05; Fig. 2).

**Table 2.** Association Between Visceral Obesity Indices and DR

	BRI			LAP			VAI		
	HR	95% CI	*P* Value	HR	95% CI	*P* Value	HR	95% CI	*P* Value
Model 1	1.16	1.10–1.21	**<0.001**	1.10	1.05–1.15	**<0.001**	1.07	1.02–1.13	**0.008**
Model 2	1.14	1.08–1.20	**<0.001**	1.09	1.04–1.15	**<0.001**	1.06	1.01–1.12	**0.020**
Model 3	1.11	1.06–1.17	**<0.001**	1.08	1.03–1.13	**0.003**	1.05	1.00–1.11	**0.043**
Excluding the first 3 y of follow-up	1.13	1.07–1.19	**<0.001**	1.09	1.03–1.14	**0.002**	1.06	1.00–1.12	**0.037**

BRI, body roundness index; CI, confidence interval; HR, hazard ratio; LAP, lipid accumulation product; VAI, visceral adiposity index.

Model 1: age + gender.

Model 2: model 1 + education + TDI + smoking + alcohol.

Model 3: model 2 + ethnicity + HTN + HbA1c + duration of DM + family history of DM.

Sensitivity analysis of excluding the first 3 y of follow-up was conducted with adjustment of model 3.

The *P* values in bold represent statistical significance.

To further verify the primary results, we performed a series of subgroup analyses (Table 3). Both BRI and LAP were significantly correlated with DR in individuals over 60 years of age, whereas VAI was not. Moreover, we found a significant relationship between BRI and DR in subjects with obesity but not in those with normal BMI, whereas VAI exhibited the inverse pattern.

**Table 3.** Subgroup Analyses of the Association Between Visceral Obesity Indices and DR

	BRI			LAP			VAI		
	HR	95% CI	*P* Value	HR	95% CI	*P* Value	HR	95% CI	*P* Value
Age									
Age < 60 y	1.15	1.07–1.24	**<0.001**	1.05	0.98–1.13	0.181	1.02	0.95–1.10	0.57
Age ≥ 60 y	1.09	1.01–1.16	**0.02**	1.08	1.01–1.15	**0.021**	1.06	0.99–1.13	0.105
Sex									
Male	1.12	1.04–1.20	**0.002**	1.07	1.00–1.14	**0.04**	1.06	0.99–1.13	0.09
Female	1.11	1.03–1.19	**0.006**	1.06	0.98–1.15	0.167	1.02	0.95–1.11	0.56
Ethnicity									
White	1.14	1.08–1.20	**<0.001**	1.07	1.02–1.13	**0.007**	1.04	0.98–1.10	0.16
Non-White	0.94	0.81–1.10	0.43	1.06	0.90–1.24	0.50	1.07	0.94–1.22	0.294
Duration of DM									
Duration < 5 y	1.11	1.00–1.23	**0.043**	1.16	1.06–1.28	**0.002**	1.15	1.05–1.25	**0.002**
Duration ≥ 5 y	1.08	1.02–1.14	**0.012**	1.01	0.95–1.07	0.756	0.98	0.92–1.05	0.581
BMI									
Normal	0.81	0.52–1.25	0.332	1.36	0.89–2.07	0.151	1.28	1.07–1.53	**0.006**
Overweight	1.02	0.83–1.25	0.9	0.92	0.81–1.05	0.237	0.98	0.89–1.08	0.633
Obesity	1.16	1.07–1.24	**<0.001**	1.05	0.98–1.11	0.16	1.00	0.94–1.08	0.89
HTN									
HTN	1.12	1.06–1.18	**<0.001**	1.04	0.99–1.09	0.15	1.01	0.96–1.07	0.691
Non-HTN	1.06	0.91–1.24	0.430	1.16	1.01–1.33	**0.033**	1.17	1.03–1.33	**0.015**

BMI, body mass index; CI, confidence interval; DM, diabetes; HTN, hypertension; HR, hazard ratio.

The *P* values in bold represent statistical significance.

Similar results were found in sensitivity analyses that excluded DR onset within the first 3 years of follow-up, considered competing mortality risk, added BMI, or WHR in the fully-adjusted model, and used the data with multiple imputation, except for VAI in the Fine and Gray model (see Table 2, Supplementary Tables S10–S12).

### Metabolic Biomarkers Associated With Visceral Obesity and DR

**Figure 3.**
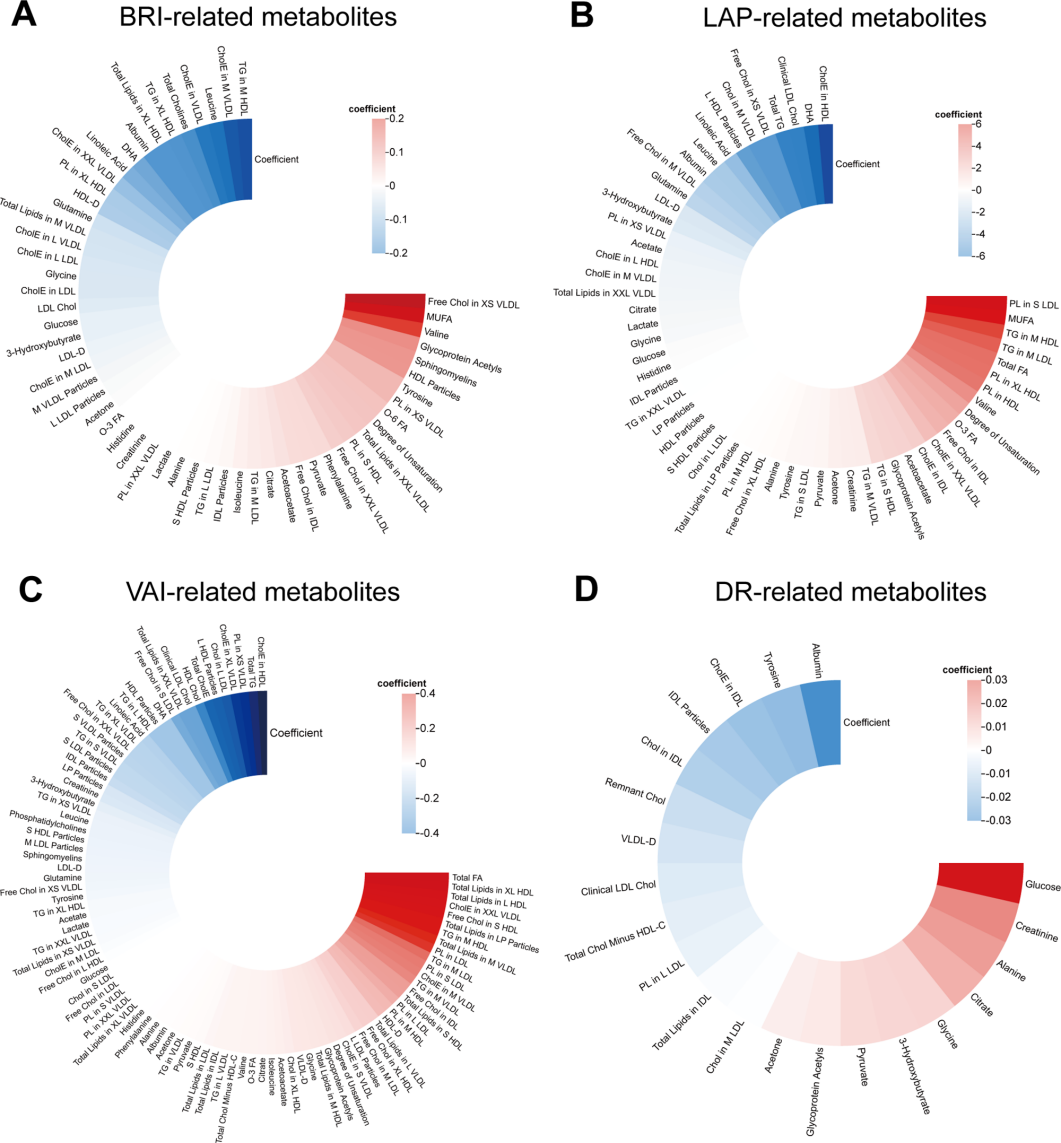
**The significant metabolites associated BRI, LAP, VAI,** **and DR.** Circles of BRI-related metabolites (**A**), LAP-related metabolites (**B**), VAI-related metabolites (**C**), and DR-related metabolites (**D**). The bars represent metabolites’ coefficients, with *red* showing coefficient > 0 and *blue* < 0. In each *circle*, metabolites were ordered according to their coefficients. Chol, cholesterol; CholE, cholesteryl esters; DHA, docosahexaenoic acid; MUFA, monounsaturated fatty acids; FA, fatty acids; PL, phospholipids; XS, very small; S, small; M, medium; L, large; XL, very large; XXL, extremely large; VLDL, very low density lipoprotein; LDL, low density lipoprotein; IDL, intermediate density lipoprotein; TG, triglycerides; O-6 FA, omega-6 fatty acids; O-3 FA, omega-3 fatty acids; LDL-D, average diameter for LDL particles; HDL-D, average diameter for HDL particles; VLDL-D, average diameter for VLDL particles; HDL-C, HDL-cholesterol; LP, lipoprotein.

**Figure 4.**
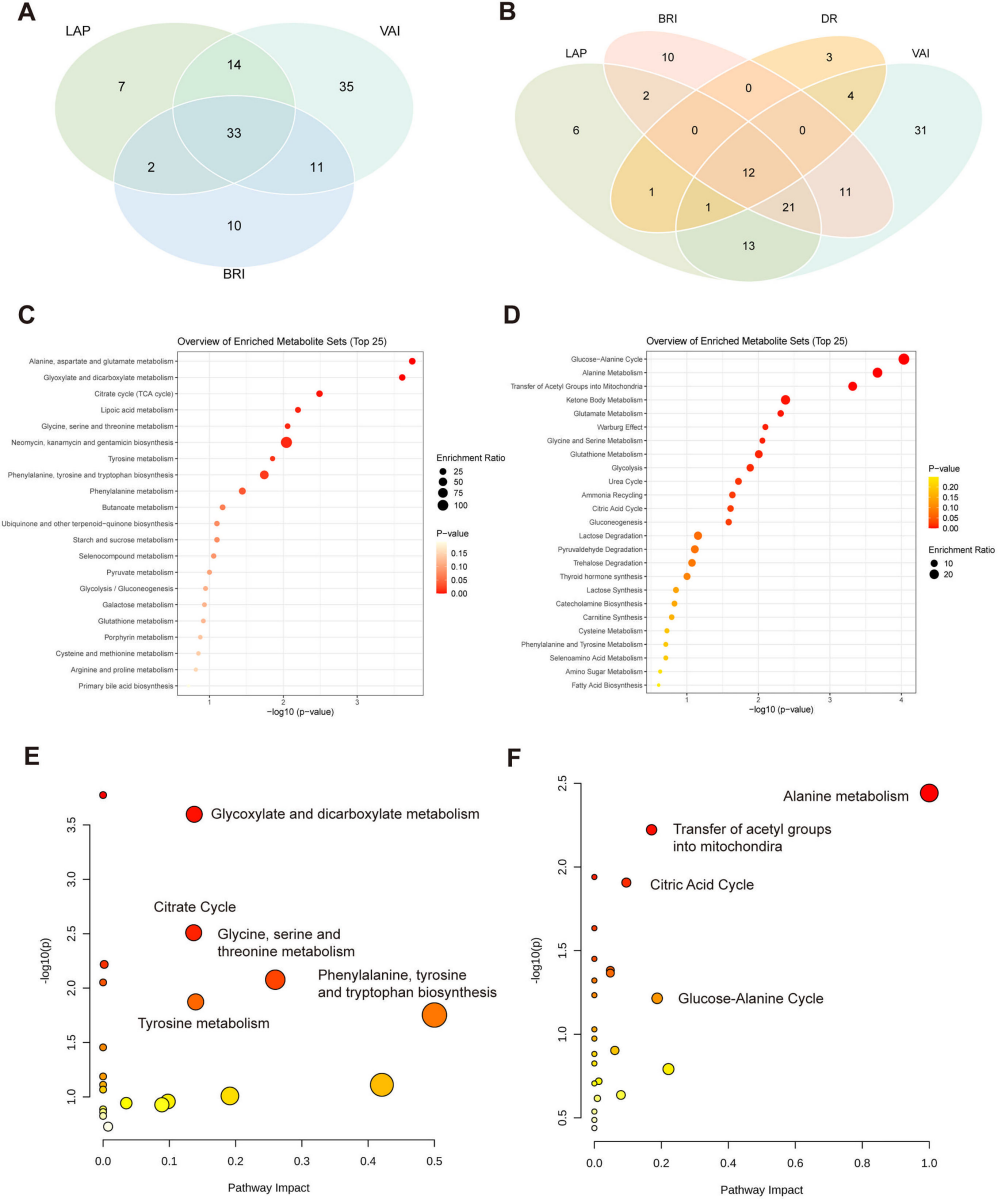
**Enrichment and pathway analysis of the intersecting metabolites.** (**A**) Venn plot showing the 33 common metabolites associated with BRI, LAP, and VAI. (**B**) Venn plot showing 12 intersected metabolites among three indices of visceral obesity and DR. Enrichment analysis of the intersected metabolites based on KEGG (**C**) and SMPDB (**D**). Pathway analysis of the intersected metabolites based on KEGG (**E**) and SMPDB (**F**).

Spearman’s correlation analysis of 168 metabolites revealed 2 essential clusters (Supplementary Fig. S1). One showed the strong relationship between TGs and total lipids, and the other demonstrated the crucial correlation between cholesterol and total lipids. EN linear regression identified 56 metabolites associated with BRI, 56 with LAP, and 93 with VAI (Figs. 3A–C). The coefficients and field ID of these metabolites are presented in Supplementary Tables S13 to S15). Monounsaturated fatty acids (MUFA) exhibited a markedly positive correlation with both BRI and LAP, whereas total fatty acids were positively associated with both LAP and VAI. The metabolite intersection across all three indices yielded 33 shared metabolites representative of visceral obesity (Fig. 4A).

EN Cox regression screened 21 metabolites with nonzero coefficients associated with DR. The most positive metabolites are glucose, creatinine, and alanine, whereas the most negative one is albumin (Fig. 3D, Supplementary Table S16). To select the metabolites correlated with both visceral obesity and DR, we overlapped the 4 groups of metabolites and found 12 intersecting biomarkers, forming a metabolic fingerprint of visceral obesity in DR (Fig. 4B, Supplementary Table S17). Among them, glucose, creatinine, alanine, citrate, glycine, and albumin were the most essential ones.

### Biological Pathway and Network Analysis

**Figure 5.**
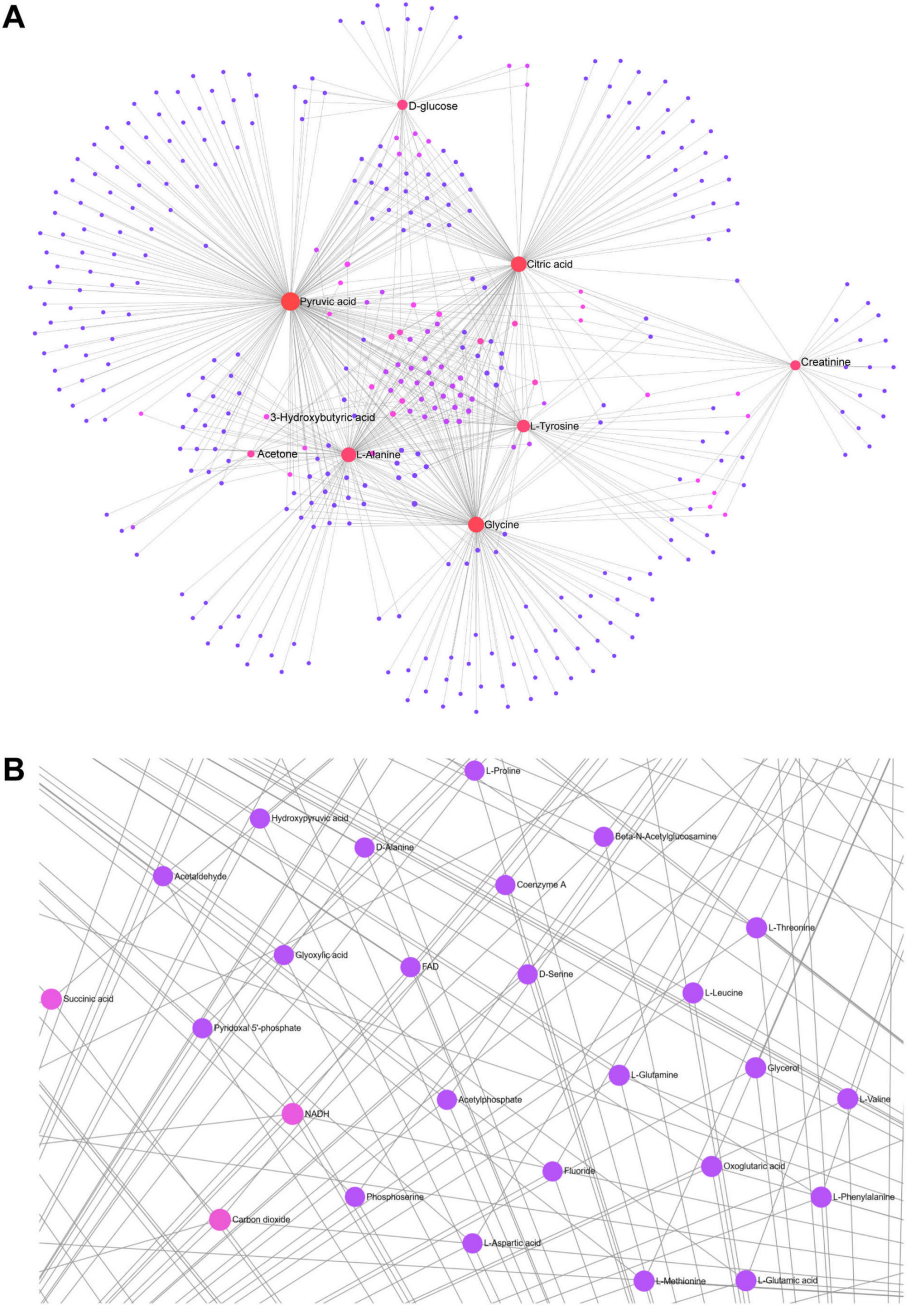
**Network analysis of the intersected metabolites.** (**A**) Metabolite-metabolite network based on 9 selected metabolites. Dots represent different metabolites. (**B**) The enlarged view of the central part of the network. Each dot shows one metabolite and lines represent the interactions between metabolites.

MetaboAnalyst online platform was used to investigate the biological pathways enriched by the 12 intersected metabolites. Three metabolites (albumin, IDL particles, and glycoprotein acetyls) were not recognized by the platform, resulting in nine metabolites for further analysis. Based on KEGG and SMPDB, we found that the intersected metabolites are mainly enriched in pathways such as alanine, aspartate and glutamate metabolism, glucose-alanine cycle, glyoxylate and dicarboxylate metabolism, citrate cycle, and ketone body metabolism (*P* < 0.05; Figs. 4C, 4D). Pathway analysis demonstrated similar results and emphasized the importance of amino acids such as alanine, glycine, serine, threonine, and phenylalanine in the visceral obesity mechanism of DR development (*P* < 0.05; Figs. 4E, 4F). Furthermore, a network was created with 374 nodes (metabolites) and 721 edges (interactions) based on the nine core metabolites (Fig. 5A). Zooming in on the network, we detected several mediating metabolites in the central part, including nicotinamide adenine dinucleotide hydrogen (NADH) and succinic acid (Fig. 5B).

## Discussion

In this prospective cohort study of approximately 15,000 participants with DM, we found that the 3 novel indices of visceral obesity were positively associated with incident DR, regardless of lifestyle, sociodemographic, and diabetic risk factors during a follow-up of 10 years. Further, using high-throughput metabolomic data, we identified four groups of metabolites associated with BRI, LAP, VAI, and DR, respectively. The core metabolites shared by visceral obesity and DR were highly enriched in the metabolism of alanine, glutamate, and glycine. We also discovered the interaction among these core metabolites through energy-related metabolites such as succinic acid and NADH.

Our findings aligned with previous studies. A cross-sectional study recruiting over 2000 subjects with DM identified that the increment of LAP and VAI correlated with a higher DR likelihood.^40^ Another Asian study of a 2-year follow-up presented similar outcomes except for a nonlinear dose-response curve between LAP or VAI and DR, which was inconsistent with our study.^41^ This may be due to the discrepancy between Asians and Europeans. Compared with Europeans, South Asians tended to have more severe DR in less time.^42^ Besides, Asians had a higher prevalence of visceral obesity and lower insulin sensitivity than Europeans with the same BMI.^43^ As shown above, previous studies on the association between visceral obesity indices and DR included limited ethnic diversity and were small-scale, cross-sectional, or longitudinal with short visiting time. Therefore, our 10-year longitudinal study of approximately 15,000 participants from multiple racial backgrounds offered stronger evidence of the relationship between visceral fat and DR.

We first discovered the relationship between BRI and DR, and this index was previously reported to correlate with all-cause mortality.^44^ BRI demonstrated consistent DR associations across nearly all subgroups, suggesting its potential as an independent predictive marker. Moreover, we found that the association between VAI and DR was not significant in the Fine and Gray model, suggesting the underlying competing effect of death on DR risk.

One of the most prominent advantages of this research is using metabolomic profiling to explore the association between visceral obesity and DR, whereas most preceding studies solely focused on superficial correlations. Mechanistically, excessive fat contributes to a constellation of metabolic disturbances, including dyslipidemia and β-cell dysfunction.^45^^–^^47^ From 168 types of plasma metabolites, we identified 12 core metabolites related to visceral obesity and DR. In addition to established markers such as glucose and creatinine, we also discovered new DR-related biomarkers such as 3-hydroxybutyrate, glycine, tyrosine, and alanine. Emerging evidence has also supported their roles in DM or DR. For instance, 3-hydroxybutyrate weakened insulin resistance by suppressing peroxisome proliferator-activated receptor γ (PPARγ) Ser273 phosphorylation in adipocytes, showing a protective effect in type 2 diabetes (T2D).^48^ Gregory et al. discovered that glycine accumulated more in the vitreous humor of subjects with proliferative diabetic retinopathy (PDR) compared with patients with DM without PDR.^49^ Tyrosine and alanine were related to reduced insulin secretion and a higher risk of incident DM.^50^

Our study revealed that energy metabolism may mediate the influence of core metabolites on DR development. Through network analysis, we discovered that the core metabolites interact with each other through hundreds of mediators, among which the pivotal one is succinic acid. Beyond its canonical role in the tricarboxylic acid cycle, succinic acid also acts as a hormone via succinate receptor 1, impacting osteoclastogenesis in T2D mice.^51^ A recent systematic review also reported that succinic acid was a potential marker for DR.^52^

The enrichment and pathway analyses pointed out the significance of alanine metabolism. Early research revealed that alanine aminotransferase (ALT) elevation was strongly associated with abnormalities of glucose and lipid metabolism, resulting in visceral fat accumulation and insulin resistance.^53^ Moreover, increased ALT level was directly correlated with trunk fat.^54^ Alanine was also previously identified as a candidate DR biomarker.^52^ To our knowledge, this study provides the first evidence of alanine metabolism as a mechanistic link between visceral obesity and DR development.

Our findings have far-reaching significance in public health and clinical relevance. Instead of using complicated imaging facilities, individuals can monitor their obesity status and assess DR risk by measuring visceral obesity indices routinely. This contributes to the early detection of DR and prevents vision loss, especially in rural areas where medical equipment is not widely accessible. Furthermore, identification of the metabolites related to visceral obesity and DR enables us to understand the underlying mechanism by which visceral obesity affects DR, paving the way for targeted prevention strategies.

However, this study is not exempt from limitations. First, selection bias existed, as the UKB population was generally healthy. Second, the diagnosis through ICD codes resulted in an overall late diagnosis, so early DR may be overlooked. Third, although the study included individuals of multiple races, the majority of the UKB participants are Caucasians. Thus, the association between visceral fat and DR may not be solid in other races. Last, the metabolic mechanisms discovered by this study, whereas supported by epidemiological and sequencing data, require verification through biological experiments.

## Conclusions

In conclusion, to the best of our knowledge, we conducted the largest longitudinal study investigating the correlation between visceral obesity and DR risk to date. The strongly positive association between visceral obesity and DR was demonstrated through three novel indices (BRI, LAP, and VAI). Notably, we found a metabolic fingerprint and associated pathways, among which amino acids such as alanine and energy metabolism were essential, influencing the visceral obesity mechanism of DR. Our findings underscore the necessity of the periodic monitoring of visceral obesity indices in DR prevention and the importance of plasma metabolites in the effect of visceral obesity on DR.

## Supplementary Material

Supplement 1

Supplement 2
