# Supplementary material for: Novel Visceral Obesity Indicators and Associated Metabolic Fingerprint in Incident Diabetic Retinopathy
Source: Invest Ophthalmol Vis Sci. 2025 Sep 8;66(12):17. doi: 10.1167/iovs.66.12.17 (PMC12422393; doi:10.1167/iovs.66.12.17)
Supplement: Supplement 1 [file iovs-66-12-17_s001.docx]

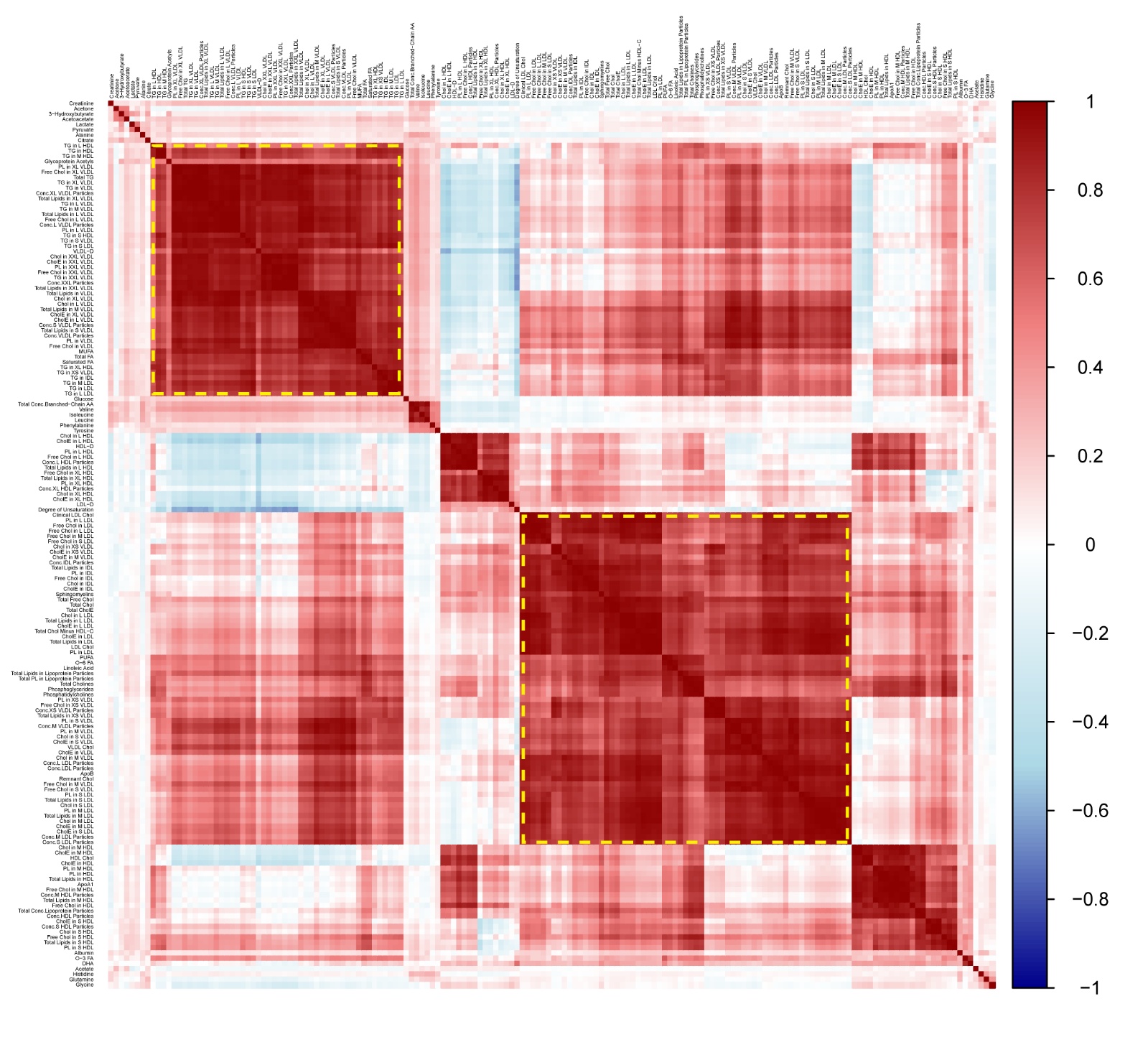


**Figure S1. Spearman’s correlation of 168 metabolic biomarkers.** Spearman’s rank correlation coefficient was shown in the bar on the right, with blue representing coefficient < 0 and red > 0. The yellow dashed bounding boxes marked the two main clusters. AA, amino acids; ApoA1, Apolipoprotein A1; ApoB, Apolipoprotein B; Conc, concentration; Chol, cholesterol; CholE, Cholesteryl Esters; DHA, Docosahexaenoic Acid; MUFA, Monounsaturated Fatty Acids; PUFA, Polyunsaturated Fatty Acids; FA, Fatty Acids; PL, Phospholipids; XS, Very Small; S, Small; M, Medium; L, Large; XL, Very Large; XXL, Extremely Large; VLDL, Very Low Density Lipoprotein; LDL, Low Density Lipoprotein; IDL, Intermediate Density Lipoprotein; TG, Triglycerides; O-6 FA, Omega-6 Fatty Acids; O-3 FA, Omega-3 Fatty Acids; LDL-D, Average Diameter for LDL Particles; HDL-D, Average Diameter for HDL Particles; VLDL-D, Average Diameter for VLDL Particles
