# Supplementary material for: Novel Visceral Obesity Indicators and Associated Metabolic Fingerprint in Incident Diabetic Retinopathy
Source: Invest Ophthalmol Vis Sci. 2025 Sep 8;66(12):17. doi: 10.1167/iovs.66.12.17 (PMC12422393; doi:10.1167/iovs.66.12.17)
Supplement: Supplement 2 [file iovs-66-12-17_s002.docx]

**Table S1. Diagnosis of diabetes mellitus**

| **Source** | **Field ID** | **Definition of field ID** | **code** |
| --- | --- | --- | --- |
| Verbal interview | 20002 | Non-cancer illness code, self-reported | 1220,1222  1223,1221 |
|  | 20003 | Treatment/medication code | 1140883066  1140884600  1141189090  1140874718  1141152590  1140874646  1141157284  1140874744  1140874658  1141168660  1141173882  1141177600  1141171646  1140868902  1140861942  1141180722 |
| Touchscreen questionnaire | 2443 | Diabetes diagnosed by doctor | 1 |
|  | 6153 | Medication for cholesterol, blood pressure, diabetes, or take exogenous hormones | 3 |
|  | 6177 | Medication for cholesterol, blood pressure or diabetes | 3 |
| Hospital inpatients | 41270 | Diagnoses - ICD10 | E100-119, E121, E123, E125, E128, E129, E130-139, E140-149 |
| First occurrence | 130708 | Date E11 first reported  (non-insulin-dependent diabetes mellitus) |  |
|  | 130706 | Date E10 first reported  (insulin-dependent diabetes mellitus) |  |
|  | 130710 | Date E12 first reported  (malnutrition-related diabetes mellitus) |  |
|  | 130712 | Date E13 first reported  (other specified diabetes mellitus) |  |
|  | 130714 | Date E14 first reported  (unspecified diabetes mellitus) |  |
| Blood chemistry | 30740 | Glucose |  |
|  | 30750 | Glycated hemoglobin (HbA1c) |  |

**Table S2. Diagnosis of diabetic retinopathy**

| **Source** | **Field ID** | **Definition of field ID** | **code** |
| --- | --- | --- | --- |
| Verbal interview | 20002 | Non-cancer illness code, self-reported | 1276 |
| Touchscreen questionnaire | 6148 | Eye problems/disorders | 1 |
| Hospital inpatients | 41270 | Diagnoses - ICD10 | H36.0, E10.3, E11.3, E13.3, E14.3 |
| First occurrence | 131184 | Date H36 first reported (retinal disorders in diseases classified elsewhere) |  |

**Table S3. 168 types of directly-measured metabolites**

| **Cholesterol concentrations** |
| --- |
| Cholesterol in Chylomicrons and Extremely Large VLDL  Cholesterol in IDL  Cholesterol in Large HDL  Cholesterol in Large LDL  Cholesterol in Large VLDL  Cholesterol in Medium HDL  Cholesterol in Medium LDL  Cholesterol in Medium VLDL  Cholesterol in Small HDL  Cholesterol in Small LDL  Cholesterol in Small VLDL  Cholesterol in Very Large HDL  Cholesterol in Very Large VLDL  Cholesterol in Very Small VLDL  Clinical LDL Cholesterol  HDL Cholesterol  LDL Cholesterol  VLDL Cholesterol  Total Cholesterol  Total Cholesterol Minus HDL-C  Remnant Cholesterol (Non-HDL, Non-LDL -Cholesterol) |
| **Esterified cholesterol concentrations** |
| Cholesteryl Esters in Chylomicrons and Extremely Large VLDL  Cholesteryl Esters in HDL  Cholesteryl Esters in IDL  Cholesteryl Esters in LDL  Cholesteryl Esters in Large HDL  Cholesteryl Esters in Large LDL  Cholesteryl Esters in Large VLDL  Cholesteryl Esters in Medium HDL  Cholesteryl Esters in Medium LDL  Cholesteryl Esters in Medium VLDL  Cholesteryl Esters in Small HDL  Cholesteryl Esters in Small LDL  Cholesteryl Esters in Small VLDL  Cholesteryl Esters in VLDL  Cholesteryl Esters in Very Large HDL  Cholesteryl Esters in Very Large VLDL  Cholesteryl Esters in Very Small VLDL  Total Esterified Cholesterol |
| **Lipoprotein particle concentrations** |
| Concentration of Chylomicrons and Extremely Large VLDL Particles  Concentration of HDL Particles  Concentration of IDL Particles  Concentration of LDL Particles  Concentration of Large HDL Particles  Concentration of Large LDL Particles  Concentration of Large VLDL Particles  Concentration of Medium HDL Particles  Concentration of Medium LDL Particles  Concentration of Medium VLDL Particles  Concentration of Small HDL Particles  Concentration of Small LDL Particles  Concentration of Small VLDL Particles  Concentration of VLDL Particles  Concentration of Very Large HDL Particles  Concentration of Very Large VLDL Particles  Concentration of Very Small VLDL Particles  Total Concentration of Lipoprotein Particles |
| **Free cholesterol concentrations** |
| Free Cholesterol in Chylomicrons and Extremely Large VLDL  Free Cholesterol in HDL  Free Cholesterol in IDL  Free Cholesterol in LDL  Free Cholesterol in Large HDL  Free Cholesterol in Large LDL  Free Cholesterol in Large VLDL  Free Cholesterol in Medium HDL  Free Cholesterol in Medium LDL  Free Cholesterol in Medium VLDL  Free Cholesterol in Small HDL  Free Cholesterol in Small LDL  Free Cholesterol in Small VLDL  Free Cholesterol in VLDL  Free Cholesterol in Very Large HDL  Free Cholesterol in Very Large VLDL  Free Cholesterol in Very Small VLDL  Total Free Cholesterol |
| **Phospholipid concentrations** |
| Phospholipids in Chylomicrons and Extremely Large VLDL  Phospholipids in HDL  Phospholipids in IDL  Phospholipids in LDL  Phospholipids in Large HDL  Phospholipids in Large LDL  Phospholipids in Large VLDL  Phospholipids in Medium HDL  Phospholipids in Medium LDL  Phospholipids in Medium VLDL  Phospholipids in Small HDL  Phospholipids in Small LDL  Phospholipids in Small VLDL  Phospholipids in VLDL  Phospholipids in Very Large HDL  Phospholipids in Very Large VLDL  Phospholipids in Very Small VLDL  Total Phospholipids in Lipoprotein Particles |
| **Total lipid concentrations** |
| Total Lipids in Chylomicrons and Extremely Large VLDL  Total Lipids in HDL  Total Lipids in IDL  Total Lipids in LDL  Total Lipids in Large HDL  Total Lipids in Large LDL  Total Lipids in Large VLDL  Total Lipids in Lipoprotein Particles  Total Lipids in Medium HDL  Total Lipids in Medium LDL  Total Lipids in Medium VLDL  Total Lipids in Small HDL  Total Lipids in Small LDL  Total Lipids in Small VLDL  Total Lipids in VLDL  Total Lipids in Very Large HDL  Total Lipids in Very Large VLDL  Total Lipids in Very Small VLDL |
| **Triglyceride concentrations** |
| Triglycerides in Chylomicrons and Extremely Large VLDL  Triglycerides in HDL  Triglycerides in IDL  Triglycerides in LDL  Triglycerides in Large HDL  Triglycerides in Large LDL  Triglycerides in Large VLDL  Triglycerides in Medium HDL  Triglycerides in Medium LDL  Triglycerides in Medium VLDL  Triglycerides in Small HDL  Triglycerides in Small LDL  Triglycerides in Small VLDL  Triglycerides in VLDL  Triglycerides in Very Large HDL  Triglycerides in Very Large VLDL  Triglycerides in Very Small VLDL  Total Triglycerides |
| **Fatty acids** |
| Total Fatty Acids  Degree of Unsaturation  Omega-3 Fatty Acids  Omega-6 Fatty Acids  Polyunsaturated Fatty Acids  Monounsaturated Fatty Acids  Saturated Fatty Acids  Linoleic Acid  Docosahexaenoic Acid |
| **Amino acids** |
| Alanine  Glutamine  Glycine  Histidine  Total Concentration of Branched-Chain Amino Acids (Leucine + Isoleucine + Valine)  Isoleucine  Leucine  Valine  Phenylalanine  Tyrosine |
| **Glycolysis-related metabolites** |
| Lactate  Glucose  Citrate  Pyruvate |
| **Ketone bodies & fluid balance** |
| 3-Hydroxybutyrate  Acetate  Acetoacetate  Acetone  Albumin  Creatinine  Glycoprotein Acetyls |
| **Apolipoproteins & lipoprotein particle sizes & other lipids** |
| Apolipoprotein A1  Apolipoprotein B  Average Diameter for HDL Particles  Average Diameter for LDL Particles  Average Diameter for VLDL Particles  Phosphoglycerides  Total Cholines  Phosphatidylcholines  Sphingomyelins |

**Table S4. The number of missing values and their proportions**

| **Characteristics** | **Missing Count** | **Missing Ratio** |
| --- | --- | --- |
| Townsend index | 20 | 0.14% |
| Smoking | 153 | 1.04% |
| Alcohol | 75 | 0.51% |
| HbA1c | 680 | 4.61% |
| Duration of diabetes | 3676 | 24.94% |
| Glucose | 1871 | 12.70% |
| Serum creatinine | 688 | 4.67% |
| HDL cholesterol | 1871 | 12.70% |
| LDL | 725 | 4.92% |
| Triglycerides | 708 | 4.80% |
| BMI | 109 | 0.74% |
| WHR | 54 | 0.37% |
| BRI | 88 | 0.60% |
| LAP | 756 | 5.13% |
| VAI | 1929 | 13.09% |

*Only the covariates with missing values were shown in this table and those with complete data were not shown.

HDL, high-density lipoprotein; LDL, low-density lipoprotein; BMI, body mass index; WHR, waist-to-hip ratio; BRI, body roundness index; LAP, lipid accumulation product; VAI, visceral adiposity index.

**Table S5. Baseline characteristics of the subjects without data imputation**

| **Characteristics**  **Mean (SD) or n (%)** | **Non DR**  **(N=7,883)** | **DR**  **(N=1,102)** | ***P*** |
| --- | --- | --- | --- |
| Age, years, mean (SD) | 59.76 (7.08) | 60.41 (6.89) | 0.004 |
| Male, n (%) | 5104 (64.7) | 674 (61.2) | 0.022 |
| College or university degree, n (%) | 1878 (23.8) | 261 (23.7) | 0.949 |
| Townsend Deprivation Index, mean (SD) | -0.66 (3.31) | -0.50 (3.40) | 0.124 |
| Smoking, n (%) |  |  | 0.853 |
| Never | 3503 (44.4) | 489 (44.4) |  |
| Previous | 3536 (44.9) | 489 (44.4) |  |
| Current | 844 (10.7) | 124 (11.3) |  |
| Alcohol, n (%) |  |  | 0.181 |
| Never | 607 (7.7) | 101 (9.2) |  |
| Previous | 512 (6.5) | 77 (7.0) |  |
| Current | 6764 (85.8) | 924 (83.8) |  |
| Follow-up time, years, mean (SD) | 12.76 (2.76) | 9.07 (3.69) | <0.001 |
| Diabetes-related factors |  |  |  |
| White, n (%) | 7157 (90.8) | 972 (88.2) | 0.007 |
| HTN, n (%) | 6316 (80.1) | 973 (88.3) | <0.001 |
| HbA1c, mmol/mol, mean (SD) | 51.31 (12.67) | 57.76 (15.20) | <0.001 |
| Glucose, mmol/L, mean (SD) | 7.29 (3.03) | 8.57 (3.94) | <0.001 |
| Duration of diabetes, years, mean (SD) | 7.36 (9.50) | 10.85 (11.15) | <0.001 |
| Family history of diabetes, n (%) | 3145 (39.9) | 458 (41.6) | 0.306 |
| Diabetes medication use, n (%) |  |  |  |
| Oral hypoglycemic drugs | 4520 (57.3) | 738 (67.0) | <0.001 |
| Cholesterol-lowering drugs | 5928 (75.2) | 871 (79.0) | 0.006 |
| Hypotensive drugs | 4933 (62.6) | 734 (66.6) | 0.010 |
| Insulin use | 1084 (13.8) | 346 (31.4) | <0.001 |
| Blood biochemistry markers, mean (SD) |  |  |  |
| Serum creatinine, umol/L | 74.89 (19.37) | 77.04 (30.85) | 0.002 |
| HDL cholesterol, mmol/L | 1.19 (0.32) | 1.19 (0.32) | 0.894 |
| LDL, mmol/L | 2.71 (0.75) | 2.67 (0.75) | 0.083 |
| Triglycerides, mmol/L | 2.15 (1.22) | 2.16 (1.35) | 0.839 |
| Obesity indices, mean (SD) |  |  |  |
| BMI | 31.46 (5.79) | 31.68 (5.91) | 0.231 |
| WHR | 0.95 (0.08) | 0.95 (0.09) | 0.253 |
| BRI | 5.73 (1.92) | 5.90 (2.04) | 0.006 |
| LAP | 90.37 (64.08) | 93.57 (71.80) | 0.126 |
| VAI | 3.12 (2.27) | 3.22 (2.59) | 0.178 |

DR, diabetic retinopathy; HTN, hypertension; HDL, high-density lipoprotein; LDL, low-density lipoprotein; BMI, body mass index; WHR, waist-to-hip ratio; BRI, body roundness index; LAP, lipid accumulation product; VAI, visceral adiposity index.

Data are presented as the means±standard deviations (SDs), numbers and (percentages)

**Table S6. Baseline characteristics of the subjects for BRI analysis**

| **Characteristics**  **Mean (SD) or n (%)** | **Non DR**  **(N=13,073)** | **DR**  **(N=1,577)** | ***P*** |
| --- | --- | --- | --- |
| Age, years, mean (SD) | 59.01 (7.47) | 60.21 (6.88) | <0.001 |
| Male, n (%) | 7860 (60.1) | 935 (59.3) | 0.541 |
| College or university degree, n (%) | 3129 (23.9) | 348 (22.1) | 0.106 |
| Townsend Deprivation Index, mean (SD) | -0.58 (3.36) | -0.37 (3.45) | 0.022 |
| Smoking, n (%) |  |  | 0.723 |
| Never | 6109 (46.7) | 723 (45.8) |  |
| Previous | 5482 (41.9) | 678 (43.0) |  |
| Current | 1482 (11.3) | 176 (11.2) |  |
| Alcohol, n (%) |  |  | 0.232 |
| Never | 1083 (8.3) | 150 (9.5) |  |
| Previous | 863 (6.6) | 107 (6.8) |  |
| Current | 11127 (85.1) | 1320 (83.7) |  |
| Follow-up time, years, mean (SD) | 12.81 (2.71) | 9.04 (3.73) | <0.001 |
| Diabetes-related factors |  |  |  |
| White, n (%) | 11620 (88.9) | 1350 (85.6) | <0.001 |
| HTN, n (%) | 9929 (76.0) | 1383 (87.7) | <0.001 |
| HbA1c, mmol/mol, mean (SD) | 50.81 (14.09) | 57.79 (15.46) | <0.001 |
| Glucose, mmol/L, mean (SD) | 7.09 (2.95) | 8.32 (3.96) | <0.001 |
| Duration of diabetes, years, mean (SD) | 6.78 (8.25) | 9.99 (10.34) | <0.001 |
| Family history of diabetes, n (%) | 4945 (37.8) | 648 (41.1) | 0.013 |
| Diabetes medication use, n (%) |  |  |  |
| Oral hypoglycemic drugs | 5845 (44.7) | 970 (61.5) | <0.001 |
| Cholesterol-lowering drugs | 8301 (63.5) | 1166 (73.9) | <0.001 |
| Hypotensive drugs | 7285 (55.7) | 994 (63.0) | <0.001 |
| Insulin use | 1483 (11.3) | 444 (28.2) | <0.001 |
| Blood biochemistry markers, mean (SD) |  |  |  |
| Serum creatinine, umol/L | 73.98 (18.56) | 76.30 (32.15) | <0.001 |
| HDL cholesterol, mmol/L | 1.20 (0.30) | 1.19 (0.30) | 0.171 |
| LDL, mmol/L | 2.90 (0.85) | 2.74 (0.80) | <0.001 |
| Triglycerides, mmol/L | 2.18 (1.25) | 2.22 (1.35) | 0.190 |
| Obesity indices, mean (SD) |  |  |  |
| BMI | 31.36 (5.86) | 31.80 (5.91) | 0.005 |
| WHR | 0.94 (0.09) | 0.95 (0.09) | <0.001 |
| BRI | 5.68 (1.95) | 5.95 (2.05) | <0.001 |
| LAP | 91.13 (66.07) | 97.02 (74.24) | 0.001 |
| VAI | 3.17 (2.35) | 3.32 (2.69) | 0.024 |

DR, diabetic retinopathy; HTN, hypertension; HDL, high-density lipoprotein; LDL, low-density lipoprotein; BMI, body mass index; WHR, waist-to-hip ratio; BRI, body roundness index; LAP, lipid accumulation product; VAI, visceral adiposity index.

Data are presented as the means±standard deviations (SDs), numbers and (percentages)

**Table S7. Baseline characteristics of the subjects for LAP analysis**

| **Characteristics**  **Mean (SD) or n (%)** | **Non DR**  **(N=12,467)** | **DR**  **(N=1,515)** | ***P*** |
| --- | --- | --- | --- |
| Age, years, mean (SD) | 59.00 (7.47) | 60.18 (6.93) | <0.001 |
| Male, n (%) | 7534 (60.4) | 893 (58.9) | 0.276 |
| College or university degree, n (%) | 2985 (23.9) | 332 (21.9) | 0.085 |
| Townsend Deprivation Index, mean (SD) | -0.58 (3.36) | -0.34 (3.47) | 0.011 |
| Smoking, n (%) |  |  | 0.929 |
| Never | 5799 (46.5) | 697 (46.0) |  |
| Previous | 5241 (42.0) | 644 (42.5) |  |
| Current | 1427 (11.4) | 174 (11.5) |  |
| Alcohol, n (%) |  |  | 0.101 |
| Never | 1028 (8.2) | 148 (9.8) |  |
| Previous | 825 (6.6) | 106 (7.0) |  |
| Current | 10614 (85.1) | 1261 (83.2) |  |
| Follow-up time, years, mean (SD) | 12.82 (2.71) | 9.09 (3.71) | <0.001 |
| Diabetes-related factors |  |  |  |
| White, n (%) | 11077 (88.9) | 1294 (85.4) | <0.001 |
| HTN, n (%) | 9461 (75.9) | 1328 (87.7) | <0.001 |
| HbA1c, mmol/mol, mean (SD) | 50.76 (14.09) | 57.76 (15.49) | <0.001 |
| Glucose, mmol/L, mean (SD) | 7.12 (3.01) | 8.40 (4.02) | <0.001 |
| Duration of diabetes, years, mean (SD) | 6.78 (8.24) | 9.99 (10.43) | <0.001 |
| Family history of diabetes, n (%) | 4722 (37.9) | 621 (41.0) | 0.020 |
| Diabetes medication use, n (%) |  |  |  |
| Oral hypoglycemic drugs | 5575 (44.7) | 934 (61.7) | <0.001 |
| Cholesterol-lowering drugs | 7905 (63.4) | 1115 (73.6) | <0.001 |
| Hypotensive drugs | 6933 (55.6) | 951 (62.8) | <0.001 |
| Insulin use | 1401 (11.2) | 427 (28.2) | <0.001 |
| Blood biochemistry markers, mean (SD) |  |  |  |
| Serum creatinine, umol/L | 74.09 (18.97) | 76.54 (32.86) | <0.001 |
| HDL cholesterol, mmol/L | 1.20 (0.31) | 1.19 (0.31) | 0.138 |
| LDL, mmol/L | 2.90 (0.87) | 2.74 (0.81) | <0.001 |
| Triglycerides, mmol/L | 2.19 (1.28) | 2.24 (1.38) | 0.207 |
| Obesity indices, mean (SD) |  |  |  |
| BMI | 31.36 (5.86) | 31.81 (5.90) | 0.005 |
| WHR | 0.94 (0.09) | 0.95 (0.09) | <0.001 |
| BRI | 5.68 (1.95) | 5.95 (2.05) | <0.001 |
| LAP | 91.19 (66.12) | 96.98 (74.21) | 0.002 |
| VAI | 3.17 (2.35) | 3.33 (2.69) | 0.020 |

DR, diabetic retinopathy; HTN, hypertension; HDL, high-density lipoprotein; LDL, low-density lipoprotein; BMI, body mass index; WHR, waist-to-hip ratio; BRI, body roundness index; LAP, lipid accumulation product; VAI, visceral adiposity index.

Data are presented as the means±standard deviations (SDs), numbers and (percentages)

**Table S8. Baseline characteristics of the subjects for VAI analysis**

| **Characteristics**  **Mean (SD) or n (%)** | **Non DR**  **(N=11,421)** | **DR**  **(N=1,388)** | ***P*** |
| --- | --- | --- | --- |
| Age, years, mean (SD) | 58.98 (7.46) | 60.23 (6.93) | <0.001 |
| Male, n (%) | 6947 (60.8) | 823 (59.3) | 0.283 |
| College or university degree, n (%) | 2741 (24.0) | 309 (22.3) | 0.161 |
| Townsend Deprivation Index, mean (SD) | -0.60 (3.35) | -0.31 (3.48) | 0.003 |
| Smoking, n (%) |  |  | 0.869 |
| Never | 5292 (46.3) | 633 (45.6) |  |
| Previous | 4810 (42.1) | 591 (42.6) |  |
| Current | 1319 (11.5) | 164 (11.8) |  |
| Alcohol, n (%) |  |  | 0.058 |
| Never | 935 (8.2) | 137 (9.9) |  |
| Previous | 744 (6.5) | 99 (7.1) |  |
| Current | 9742 (85.3) | 1152 (83.0) |  |
| Follow-up time, years, mean (SD) | 12.83 (2.69) | 9.10 (3.70) | <0.001 |
| Diabetes-related factors |  |  |  |
| White, n (%) | 10169 (89.0) | 1187 (85.5) | <0.001 |
| HTN, n (%) | 8648 (75.7) | 1216 (87.6) | <0.001 |
| HbA1c, mmol/mol, mean (SD) | 50.82 (14.16) | 57.62 (15.50) | <0.001 |
| Glucose, mmol/L, mean (SD) | 7.20 (3.13) | 8.59 (4.14) | <0.001 |
| Duration of diabetes, years, mean (SD) | 6.75 (8.24) | 9.92 (10.41) | <0.001 |
| Family history of diabetes, n (%) | 4315 (37.8) | 562 (40.5) | 0.053 |
| Diabetes medication use, n (%) |  |  |  |
| Oral hypoglycemic drugs | 5105 (44.7) | 850 (61.2) | <0.001 |
| Cholesterol-lowering drugs | 7238 (63.4) | 1017 (73.3) | <0.001 |
| Hypotensive drugs | 6336 (55.5) | 876 (63.1) | <0.001 |
| Insulin use | 1270 (11.1) | 392 (28.2) | <0.001 |
| Blood biochemistry markers, mean (SD) |  |  |  |
| Serum creatinine, umol/L | 74.05 (18.85) | 76.89 (33.94) | <0.001 |
| HDL cholesterol, mmol/L | 1.20 (0.32) | 1.19 (0.32) | 0.138 |
| LDL, mmol/L | 2.91 (0.88) | 2.75 (0.81) | <0.001 |
| Triglycerides, mmol/L | 2.20 (1.28) | 2.22 (1.37) | 0.438 |
| Obesity indices, mean (SD) |  |  |  |
| BMI | 31.41 (5.88) | 31.72 (5.87) | 0.059 |
| WHR | 0.94 (0.09) | 0.95 (0.09) | 0.001 |
| BRI | 5.69 (1.95) | 5.93 (2.04) | <0.001 |
| LAP | 91.53 (66.30) | 95.85 (72.99) | 0.023 |
| VAI | 3.17 (2.35) | 3.33 (2.69) | 0.020 |

DR, diabetic retinopathy; HTN, hypertension; HDL, high-density lipoprotein; LDL, low-density lipoprotein; BMI, body mass index; WHR, waist-to-hip ratio; BRI, body roundness index; LAP, lipid accumulation product; VAI, visceral adiposity index.

Data are presented as the means±standard deviations (SDs), numbers and (percentages)

**Table S9. The ethnic background of the study population**

| **Ethnicity group** | **Non-DR** | **DR** | **Total** |
| --- | --- | --- | --- |
| White, n (%) | 11669 (89.6) | 1359 (10.4) | 13028 |
| Mixed, n (%) | 80 (96.4) | 3 (3.6) | 83 |
| Asian or Asian British (including Chinese), n (%) | 750 (87.0) | 112 (13.0) | 862 |
| Black or Black British, n (%) | 375 (87.0) | 56 (13.0) | 431 |
| Other ethnic groups, n (%) | 270 (80.8) | 64 (19.2) | 334 |

**Table S10. Association between visceral obesity indices and DR after the adjustment of BMI or WHR**

|  | **Model 4** | | | **Model 5** | | |
| --- | --- | --- | --- | --- | --- | --- |
|  | **HR** | **95% CI** | ***P*** | **HR** | **95% CI** | ***P*** |
| **BRI** | 1.24 | 1.11-1.38 | **<0.001** | 1.07 | 1.00-1.14 | **0.039** |
| **LAP** | 1.06 | 1.00-1.12 | 0.05 | 1.04 | 0.98-1.10 | 0.179 |
| **VAI** | 1.05 | 1.00-1.10 | 0.08 | 1.03 | 0.98-1.09 | 0.288 |

BRI, body roundness index; LAP, lipid accumulation product; VAI, visceral adiposity index; HR, hazard ratio; CI, confidence interval.

Model 4: Model 3+BMI

Model 5: Model 3+WHR

**Table S11. Association of visceral obesity indices with DR in the Fine and Gray competing risk model**

|  | **Estimate** | **Standard Error** | **Statistic** | ***P*** |
| --- | --- | --- | --- | --- |
| **BRI** | 0.0893 | 0.0262 | 3.4114 | **0.0006** |
| **LAP** | 0.0627 | 0.0259 | 2.4240 | **0.0150** |
| **VAI** | 0.0425 | 0.0276 | 1.5408 | 0.1200 |

BRI, body roundness index; LAP, lipid accumulation product; VAI, visceral adiposity index

**Table S12. Association of visceral obesity indices with DR using multiple imputation data**

|  | **BRI** | | | **LAP** | | | **VAI** | | |
| --- | --- | --- | --- | --- | --- | --- | --- | --- | --- |
|  | **HR** | **95% CI** | **P** | **HR** | **95% CI** | **P** | **HR** | **95% CI** | **P** |
| **Model 1** | 1.16 | 1.10-1.21 | **<0.001** | 1.10 | 1.05-1.15 | **<0.001** | 1.07 | 1.02-1.13 | **0.008** |
| **Model 2** | 1.14 | 1.08-1.20 | **<0.001** | 1.10 | 1.04-1.15 | **<0.001** | 1.06 | 1.01-1.12 | **0.018** |
| **Model 3** | 1.12 | 1.06-1.17 | **<0.001** | 1.07 | 1.02-1.13 | **0.005** | 1.05 | 1.00-1.11 | 0.055 |

BRI, body roundness index; LAP, lipid accumulation product; VAI, visceral adiposity index; HR, hazard ratio; CI, confidence interval.

Model 1: age+gender

Model 2: Model 1+education+TDI+smoking+alcohol

Model 3: Model 2+ethnicity+HTN+HbA1c+Duration of DM+family history of DM

**Table S13. Significant metabolites associated with BRI**

| **Field ID** | **Coefficient** | **Metabolite** |
| --- | --- | --- |
| 23521 | 1.306943 | Free Cholesterol in Very Small VLDL |
| 23447 | 0.857789 | Monounsaturated Fatty Acids |
| 23467 | 0.467983 | Valine |
| 23480 | 0.261266 | Glycoprotein Acetyls |
| 23438 | 0.24421 | Sphingomyelins |
| 23430 | 0.240527 | Concentration of HDL Particles |
| 23469 | 0.164988 | Tyrosine |
| 23518 | 0.163939 | Phospholipids in Very Small VLDL |
| 23445 | 0.136387 | Omega-6 Fatty Acids |
| 23443 | 0.132679 | Degree of Unsaturation |
| 23482 | 0.130845 | Total Lipids in Chylomicrons and Extremely Large VLDL |
| 23574 | 0.119332 | Phospholipids in Small HDL |
| 23486 | 0.109963 | Free Cholesterol in Chylomicrons and Extremely Large VLDL |
| 23468 | 0.085368 | Phenylalanine |
| 23472 | 0.082975 | Pyruvate |
| 23528 | 0.080562 | Free Cholesterol in IDL |
| 23476 | 0.065687 | Acetoacetate |
| 23473 | 0.052196 | Citrate |
| 23543 | 0.045215 | Triglycerides in Medium LDL |
| 23465 | 0.032618 | Isoleucine |
| 23523 | 0.017383 | Concentration of IDL Particles |
| 23536 | 0.008891 | Triglycerides in Large LDL |
| 23572 | 0.003731 | Concentration of Small HDL Particles |
| 23460 | 0.002919 | Alanine |
| 23471 | 0.000591 | Lactate |
| 23483 | 9.95E-05 | Phospholipids in Chylomicrons and Extremely Large VLDL |
| 23478 | -2.5E-05 | Creatinine |
| 23463 | -0.00019 | Histidine |
| 23444 | -0.00044 | Omega-3 Fatty Acids |
| 23477 | -0.00855 | Acetone |
| 23530 | -0.01174 | Concentration of Large LDL Particles |
| 23502 | -0.0222 | Concentration of Medium VLDL Particles |
| 23541 | -0.03161 | Cholesteryl Esters in Medium LDL |
| 23432 | -0.03806 | Average Diameter for LDL Particles |
| 23474 | -0.04809 | 3-Hydroxybutyrate |
| 23470 | -0.04856 | Glucose |
| 23405 | -0.06283 | LDL Cholesterol |
| 23417 | -0.07602 | Cholesteryl Esters in LDL |
| 23462 | -0.07647 | Glycine |
| 23534 | -0.07959 | Cholesteryl Esters in Large LDL |
| 23499 | -0.08436 | Cholesteryl Esters in Large VLDL |
| 23503 | -0.08812 | Total Lipids in Medium VLDL |
| 23461 | -0.09976 | Glutamine |
| 23433 | -0.16971 | Average Diameter for HDL Particles |
| 23553 | -0.17458 | Phospholipids in Very Large HDL |
| 23485 | -0.21323 | Cholesteryl Esters in Chylomicrons and Extremely Large VLDL |
| 23449 | -0.27104 | Linoleic Acid |
| 23450 | -0.29578 | Docosahexaenoic Acid |
| 23479 | -0.35801 | Albumin |
| 23552 | -0.36024 | Total Lipids in Very Large HDL |
| 23557 | -0.36111 | Triglycerides in Very Large HDL |
| 23436 | -0.38481 | Total Cholines |
| 23416 | -0.4725 | Cholesteryl Esters in VLDL |
| 23466 | -0.49471 | Leucine |
| 23506 | -0.60608 | Cholesteryl Esters in Medium VLDL |
| 23571 | -0.65701 | Triglycerides in Medium HDL |

**Table S14. Significant metabolites associated with LAP**

| **Field ID** | **Coefficient** | **Metabolite** |
| --- | --- | --- |
| 23546 | 22.54403 | Phospholipids in Small LDL |
| 23447 | 18.64679 | Monounsaturated Fatty Acids |
| 23571 | 13.36139 | Triglycerides in Medium HDL |
| 23543 | 11.38125 | Triglycerides in Medium LDL |
| 23442 | 10.0945 | Total Fatty Acids |
| 23553 | 9.885137 | Phospholipids in Very Large HDL |
| 23414 | 9.546412 | Phospholipids in HDL |
| 23467 | 7.755266 | Valine |
| 23443 | 6.3141 | Degree of Unsaturation |
| 23444 | 5.656496 | Omega-3 Fatty Acids |
| 23528 | 5.224633 | Free Cholesterol in IDL |
| 23485 | 4.529751 | Cholesteryl Esters in Chylomicrons and Extremely Large VLDL |
| 23527 | 3.304127 | Cholesteryl Esters in IDL |
| 23476 | 2.946802 | Acetoacetate |
| 23480 | 2.906481 | Glycoprotein Acetyls |
| 23578 | 2.668996 | Triglycerides in Small HDL |
| 23508 | 1.266532 | Triglycerides in Medium VLDL |
| 23478 | 1.237141 | Creatinine |
| 23477 | 1.050006 | Acetone |
| 23472 | 0.642437 | Pyruvate |
| 23550 | 0.545155 | Triglycerides in Small LDL |
| 23469 | 0.505671 | Tyrosine |
| 23460 | 0.320647 | Alanine |
| 23556 | 0.23254 | Free Cholesterol in Very Large HDL |
| 23567 | 0.152718 | Phospholipids in Medium HDL |
| 23423 | 0.057769 | Total Lipids in Lipoprotein Particles |
| 23533 | -0.00146 | Cholesterol in Large LDL |
| 23572 | -0.00214 | Concentration of Small HDL Particles |
| 23430 | -0.02488 | Concentration of HDL Particles |
| 23427 | -0.02541 | Total Concentration of Lipoprotein Particles |
| 23487 | -0.03454 | Triglycerides in Chylomicrons and Extremely Large VLDL |
| 23523 | -0.05128 | Concentration of IDL Particles |
| 23463 | -0.18456 | Histidine |
| 23470 | -0.25878 | Glucose |
| 23462 | -0.49195 | Glycine |
| 23471 | -0.5866 | Lactate |
| 23473 | -0.67949 | Citrate |
| 23482 | -0.82484 | Total Lipids in Chylomicrons and Extremely Large VLDL |
| 23506 | -0.87601 | Cholesteryl Esters in Medium VLDL |
| 23562 | -0.96871 | Cholesteryl Esters in Large HDL |
| 23475 | -1.17746 | Acetate |
| 23518 | -1.28344 | Phospholipids in Very Small VLDL |
| 23474 | -2.09342 | 3-Hydroxybutyrate |
| 23432 | -2.31516 | Average Diameter for LDL Particles |
| 23461 | -4.21987 | Glutamine |
| 23507 | -4.61163 | Free Cholesterol in Medium VLDL |
| 23479 | -5.22319 | Albumin |
| 23466 | -5.34004 | Leucine |
| 23449 | -6.54113 | Linoleic Acid |
| 23558 | -9.87945 | Concentration of Large HDL Particles |
| 23505 | -10.4554 | Cholesterol in Medium VLDL |
| 23521 | -10.4837 | Free Cholesterol in Very Small VLDL |
| 23407 | -13.0502 | Total Triglycerides |
| 23404 | -13.0669 | Clinical LDL Cholesterol |
| 23450 | -15.634 | Docosahexaenoic Acid |
| 23418 | -20.5085 | Cholesteryl Esters in HDL |

**Table S15. Significant metabolites associated with VAI**

| **Field ID** | **Coefficient** | **Metabolite** |
| --- | --- | --- |
| 23442 | 1.76635 | Total Fatty Acids |
| 23552 | 1.647565 | Total Lipids in Very Large HDL |
| 23559 | 1.474164 | Total Lipids in Large HDL |
| 23485 | 1.375381 | Cholesteryl Esters in Chylomicrons and Extremely Large VLDL |
| 23577 | 1.344513 | Free Cholesterol in Small HDL |
| 23423 | 1.19024 | Total Lipids in Lipoprotein Particles |
| 23571 | 1.186837 | Triglycerides in Medium HDL |
| 23503 | 1.045111 | Total Lipids in Medium VLDL |
| 23413 | 0.968523 | Phospholipids in LDL |
| 23543 | 0.684772 | Triglycerides in Medium LDL |
| 23546 | 0.615993 | Phospholipids in Small LDL |
| 23506 | 0.598739 | Cholesteryl Esters in Medium VLDL |
| 23508 | 0.553996 | Triglycerides in Medium VLDL |
| 23528 | 0.449973 | Free Cholesterol in IDL |
| 23573 | 0.44234 | Total Lipids in Small HDL |
| 23532 | 0.379592 | Phospholipids in Large LDL |
| 23567 | 0.321865 | Phospholipids in Medium HDL |
| 23433 | 0.311138 | Average Diameter for HDL Particles |
| 23496 | 0.220514 | Total Lipids in Large VLDL |
| 23556 | 0.215636 | Free Cholesterol in Very Large HDL |
| 23542 | 0.19336 | Free Cholesterol in Medium LDL |
| 23530 | 0.147144 | Concentration of Large LDL Particles |
| 23513 | 0.133985 | Cholesteryl Esters in Small VLDL |
| 23443 | 0.131855 | Degree of Unsaturation |
| 23480 | 0.115001 | Glycoprotein Acetyls |
| 23566 | 0.112528 | Total Lipids in Medium HDL |
| 23462 | 0.107439 | Glycine |
| 23431 | 0.105591 | Average Diameter for VLDL Particles |
| 23554 | 0.081138 | Cholesterol in Very Large HDL |
| 23476 | 0.070337 | Acetoacetate |
| 23465 | 0.061244 | Isoleucine |
| 23473 | 0.056978 | Citrate |
| 23444 | 0.054767 | Omega-3 Fatty Acids |
| 23467 | 0.05247 | Valine |
| 23401 | 0.050324 | Total Cholesterol Minus HDL-C |
| 23501 | 0.050279 | Triglycerides in Large VLDL |
| 23524 | 0.048085 | Total Lipids in IDL |
| 23425 | 0.042337 | Total Lipids in LDL |
| 23575 | 0.022154 | Cholesterol in Small HDL |
| 23472 | 0.020917 | Pyruvate |
| 23408 | 0.016642 | Triglycerides in VLDL |
| 23477 | 0.012203 | Acetone |
| 23479 | 0.011096 | Albumin |
| 23460 | 0.009794 | Alanine |
| 23468 | 0.005065 | Phenylalanine |
| 23463 | 0.001877 | Histidine |
| 23489 | -3.8E-06 | Total Lipids in Very Large VLDL |
| 23483 | -7.8E-05 | Phospholipids in Chylomicrons and Extremely Large VLDL |
| 23511 | -0.00017 | Phospholipids in Small VLDL |
| 23421 | -0.00021 | Free Cholesterol in LDL |
| 23547 | -0.00104 | Cholesterol in Small LDL |
| 23470 | -0.00173 | Glucose |
| 23563 | -0.00842 | Free Cholesterol in Large HDL |
| 23541 | -0.02105 | Cholesteryl Esters in Medium LDL |
| 23517 | -0.02615 | Total Lipids in Very Small VLDL |
| 23487 | -0.03272 | Triglycerides in Chylomicrons and Extremely Large VLDL |
| 23471 | -0.0333 | Lactate |
| 23475 | -0.03639 | Acetate |
| 23557 | -0.03986 | Triglycerides in Very Large HDL |
| 23469 | -0.04985 | Tyrosine |
| 23521 | -0.05904 | Free Cholesterol in Very Small VLDL |
| 23461 | -0.06192 | Glutamine |
| 23432 | -0.06472 | Average Diameter for LDL Particles |
| 23438 | -0.06647 | Sphingomyelins |
| 23537 | -0.07053 | Concentration of Medium LDL Particles |
| 23572 | -0.07452 | Concentration of Small HDL Particles |
| 23437 | -0.07876 | Phosphatidylcholines |
| 23466 | -0.08098 | Leucine |
| 23522 | -0.08538 | Triglycerides in Very Small VLDL |
| 23474 | -0.10713 | 3-Hydroxybutyrate |
| 23478 | -0.15345 | Creatinine |
| 23427 | -0.1612 | Total Concentration of Lipoprotein Particles |
| 23523 | -0.22849 | Concentration of IDL Particles |
| 23544 | -0.25282 | Concentration of Small LDL Particles |
| 23515 | -0.25338 | Triglycerides in Small VLDL |
| 23509 | -0.27835 | Concentration of Small VLDL Particles |
| 23486 | -0.28574 | Free Cholesterol in Chylomicrons and Extremely Large VLDL |
| 23494 | -0.29111 | Triglycerides in Very Large VLDL |
| 23449 | -0.31015 | Linoleic Acid |
| 23564 | -0.38383 | Triglycerides in Large HDL |
| 23430 | -0.38765 | Concentration of HDL Particles |
| 23450 | -0.38781 | Docosahexaenoic Acid |
| 23549 | -0.57268 | Free Cholesterol in Small LDL |
| 23482 | -0.62226 | Total Lipids in Chylomicrons and Extremely Large VLDL |
| 23404 | -0.62581 | Clinical LDL Cholesterol |
| 23406 | -0.84802 | HDL Cholesterol |
| 23415 | -1.09812 | Total Esterified Cholesterol |
| 23558 | -1.13296 | Concentration of Large HDL Particles |
| 23533 | -1.19859 | Cholesterol in Large LDL |
| 23492 | -1.58079 | Cholesteryl Esters in Very Large VLDL |
| 23518 | -1.684 | Phospholipids in Very Small VLDL |
| 23407 | -2.22737 | Total Triglycerides |
| 23418 | -3.34023 | Cholesteryl Esters in HDL |

**Table S16. Significant metabolites associated with DR**

| **Field ID** | **Coefficient** | **Metabolite** |
| --- | --- | --- |
| 23470 | 0.114254 | Glucose |
| 23478 | 0.04067 | Creatinine |
| 23460 | 0.033842 | Alanine |
| 23473 | 0.027426 | Citrate |
| 23462 | 0.014414 | Glycine |
| 23474 | 0.01392 | 3-Hydroxybutyrate |
| 23472 | 0.012702 | Pyruvate |
| 23480 | 0.007561 | Glycoprotein Acetyls |
| 23477 | 0.005722 | Acetone |
| 23540 | -0.00052 | Cholesterol in Medium LDL |
| 23524 | -0.00144 | Total Lipids in IDL |
| 23532 | -0.00632 | Phospholipids in Large LDL |
| 23401 | -0.00855 | Total Cholesterol Minus HDL-C |
| 23404 | -0.01024 | Clinical LDL Cholesterol |
| 23431 | -0.01317 | Average Diameter for VLDL Particles |
| 23402 | -0.01632 | Remnant Cholesterol (Non-HDL, Non-LDL -Cholesterol) |
| 23526 | -0.02394 | Cholesterol in IDL |
| 23523 | -0.02704 | Concentration of IDL Particles |
| 23527 | -0.03132 | Cholesteryl Esters in IDL |
| 23469 | -0.0337 | Tyrosine |
| 23479 | -0.0577 | Albumin |

**Table S17 Significant metabolites associated with both visceral obesity and DR**

| **Field ID** | **Metabolites** | **DR-related Coefficient** | **BRI-related Coefficient** | **LAP-related Coefficient** | **VAI-related Coefficient** |
| --- | --- | --- | --- | --- | --- |
| 23470 | Glucose | 0.114254 | -0.04856 | -0.25878 | -0.00173 |
| 23478 | Creatinine | 0.04067 | -2.50E-05 | 1.237141 | -0.15345 |
| 23460 | Alanine | 0.033842 | 0.002919 | 0.320647 | 0.009794 |
| 23473 | Citrate | 0.027426 | 0.052196 | -0.67949 | 0.056978 |
| 23462 | Glycine | 0.014414 | -0.07647 | -0.49195 | 0.107439 |
| 23474 | 3-Hydroxybutyrate | 0.01392 | -0.04809 | -2.09342 | -0.10713 |
| 23472 | Pyruvate | 0.012702 | 0.082975 | 0.642437 | 0.020917 |
| 23480 | Glycoprotein Acetyls | 0.007561 | 0.261266 | 2.906481 | 0.115001 |
| 23477 | Acetone | 0.005722 | -0.00855 | 1.050006 | 0.012203 |
| 23523 | Concentration of IDL Particles | -0.02704 | 0.017383 | -0.05128 | -0.22849 |
| 23469 | Tyrosine | -0.0337 | 0.164988 | 0.505671 | -0.04985 |
| 23479 | Albumin | -0.0577 | -0.35801 | -5.22319 | 0.011096 |
